# Primeiro registro de *Lutzomyia longipalpis* (Lutz & Neiva, 1912) em Macapá, Amapá

**DOI:** 10.11606/s1518-8787.2024058005963

**Published:** 2024-11-25

**Authors:** Keison de Souza Cavalcante, Allan Kardec Ribeiro Galardo, José Ferreira Saraiva, Tatiane Alves Barbosa, Érika Oliveira Galeno, Marcio Claudio de Lima Nunes, Ana Paula Sales de Andrade Correa, Nayma da Silva Picanço, Fredy Galvis-Ovallos, Eunice Aparecida Bianchi Galati

**Affiliations:** I Instituto de Pesquisas Científicas e Tecnológicas do Amapá (IEPA). Laboratório de Entomologia Médica (Labenmed). Macapá, AP, Brasil; II Universidade de São Paulo. Faculdade de Saúde Pública. Programa de Pós-graduação em Saúde Pública. São Paulo, SP, Brasil; III Secretaria Municipal de Vigilância em Saúde (SMVS). Vigilância Epidemiológica/Macapá, Vigilância Ambiental, Zoonoses, Controle de Vetores, Entomologia. Macapá, AP, Brasil; IV Universidade de São Paulo. Faculdade de Saúde Pública. Departamento de Epidemiologia. São Paulo, SP, Brasil

**Keywords:** Leishmaniose Visceral, Vetor, Vigilância Entomológica

## Abstract

*Lutzomyia longipalpis* (Lutz & Neiva, 1912) é o vetor do agente da leishmaniose visceral (LV) no Novo Mundo de maior relevância epidemiológica. Em 25 de outubro de 2023, o Centro de Informações Estratégicas em Vigilância em Saúde de Macapá detectou um caso de LV no bairro Km9 desse município. O objetivo deste estudo foi identificar as espécies de flebotomíneos nessa área a fim de auxiliar na confirmação da autoctonia do caso. Foram coletados 12 espécimes, desses, cinco foram de *Lu. longipalpis,* confirmando assim a presença do vetor do agente da LV e a possível autoctonia da transmissão.

## INTRODUÇÃO

 Os flebotomíneos pertencem à família Psychodidae, subfamília Phlebotominae que atualmente é composta por aproximadamente 1.060 espécies descritas no mundo ^1^ . Entre elas, *Lutzomyia longipalpis* (Lutz & Neiva, 1912) é o vetor do agente da leishmaniose visceral (LV) no Novo Mundo de maior relevância epidemiológica ^2^ ; apresenta ampla distribuição, do México à Argentina, e, no bioma amazônico, com exceção do Amazonas, foi registrado em todos os demais estados ^3^ . 

 A forma zoonótica da LV, causada pelo protozoário *Leishmania (Leishmania) infantum chagasi* tem como principal reservatório o cão. Essa doença é considerada crônica grave, que afeta principalmente crianças e idosos, com elevada letalidade quando não se institui o tratamento oportuno e adequado ^4^ . 

 A LV está em processo de expansão geográfica no Brasil, associado a diferentes fatores como a urbanização, a mobilidade de cães infectados e principalmente a dispersão do vetor ^4^ . Atualmente, casos de LV têm sido registrados em 25 dos 26 estados da Federação; todavia, em algumas regiões, a transmissão é associada a outras espécies de flebotomíneos, consideradas vetores secundários ^4^ . A ocorrência da LV tem sido reportada em áreas mais a leste e norte do bioma amazônico do Brasil, sendo tema de debate se é uma doença enzoótica da região ou introduzida por cães infectados migrantes ^5^ . 

 A cidade de Macapá era considerada uma área sem transmissão de LV até 2017, quando foram relatados casos caninos autóctones ^6^ . A presença de casos caninos antecede a ocorrência de casos humanos, dependendo da presença de vetores competentes na área de transmissão. Apesar do risco de estabelecimento de um ciclo urbano de transmissão dessa infecção, foram feitos poucos estudos sobre a fauna de flebotomíneos nesse município, e a presença de *Lu. longipalpis* não foi detectada ^6^^,^^7^ . 

No dia 25 de outubro de 2023, o Centro de Informações Estratégicas em Vigilância em Saúde (Cievs) de Macapá detectou um caso confirmado de LV em um paciente de 51 anos de idade residente no bairro Km9 do referido município. Para auxiliar na confirmação da autoctonia do caso, foi realizada uma investigação entomológica para detecção de flebotomíneos, cujos resultados são aqui apresentados.

## MÉTODOS

 A pesquisa entomológica foi realizada no bairro Km9, localizado em área periférica da cidade de Macapá. Nessa localidade, a aproximadamente 300 metros do local de residência do paciente, foi identificado um domicílio que serve como abrigo para aproximadamente 150 cães. As capturas foram realizadas no peridomicílio dessa propriedade (00º03’47.82’‘N 51º08’12.94’’W) ( Figura ). Foram selecionados três pontos (nas duas laterais e no fundo do terreno onde está localizado o domicílio que serve de abrigo de cães) para instalação de uma armadilha luminosa tipo CDC. As capturas foram realizadas por três dias consecutivos, no período de 1 a 3 de novembro de 2023, totalizando 108 horas de amostragem. 

 Os flebotomíneos coletados foram levados ao Laboratório de Entomologia Médica (Labenmed) do Instituto de Pesquisas Científicas e Tecnológicas do Amapá (Iepa) para triagem por sexo e identificação taxonômica, segundo Galati ^3^ . Todos os espécimes coletados foram depositados na Coleção Zoológica do Iepa, com os respectivos números de tombo. 

**Figura.**
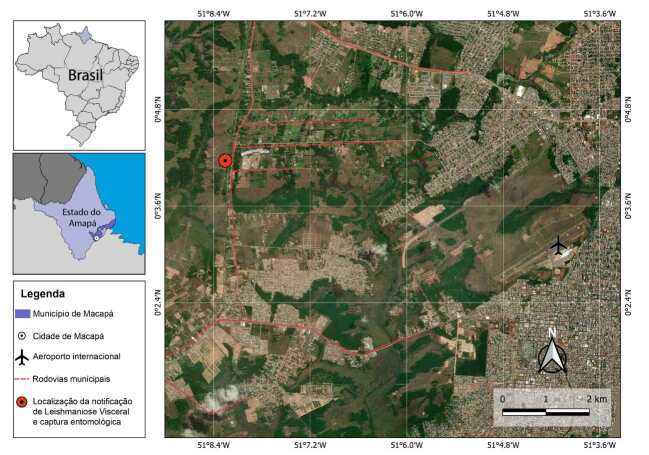
Área de estudo indicando o local da pesquisa entomológica realizada em área periférica do município de Macapá no bairro Km 9 entre os dias 1 e 3 de novembro de 2023.

## RESULTADOS E DISCUSSÃO

 Nos três dias de captura, foram coletados no peridomicílio 12 indivíduos de flebotomíneos pertencentes a seis espécies, à saber: *Lu. longipalpis* (n = 5); *Nyssomyia antunesi* (Coutinho, 1939) (n = 3); *Micropygomyia (Sauromyia) rorotaensis* (Floch & Abonnenc, 1944) (n = 1); *Psychodopygus squamiventris maripaensis* (Floch & Abonnenc, 1946) (n = 1); *Psychodopygus ayrozai* (Barretto & Coutinho, 1940) (n = 1); e *Evandromyia (Evandromyia) brachyphalla* (Mangabeira, 1941) (n = 1) ( Tabela ). 

**Tabela.** Espécies de flebotomíneos coletados em área periurbana do município de Macapá no bairro Km 9 entre os dias 1 e 3 de novembro de 2023.

**Espécie**	**Macho**	**Fêmea**	**Total**
*Lu. longipalpis*	1	4	5
*Ny. antunesi*	1	2	3
*Mi. (Sau.) rorotaensis*	–	1	1
*Ps. squamiventris maripaensis*	–	1	1
*Ps. ayrozai*	1	–	1
*Ev. (Eva.) brachyphalla*	–	1	1
**TOTAL GERAL**	**3**	**9**	**12**

 Dos 12 espécimes coletados, cinco foram de *Lu. longipalpis,* confirmando a presença do principal vetor do agente da LV no município de Macapá, confirmando a ocorrência de transmissão local da doença. 

 A não detecção da presença de *Lu. longipalpis* nas pesquisas entomológicas realizadas previamente no município ^6 , 7^ pode estar relacionada a uma introdução recente da espécie na área ou à sua baixa densidade que dificulta a detecção. No entanto, vale lembrar que a ocorrência de *Lu. longipalpis* já tinha sido registrada no município de Ferreira Gomes, localizado a 137 km de Macapá ^8^ . 

 Embora a ocorrência de leishmaniose canina tenha sido previamente reportada em Macapá, a detecção do caso humano aliada ao encontro do vetor *Lu. longipalpis* na área urbana do município alerta para a necessidade da ampla divulgação da informação para os serviços de saúde, no sentido de auxiliar na vigilância para o diagnóstico precoce de casos humanos e tratamento oportuno, além da necessidade de dimensionar o risco da infecção para a população humana e canina e a implementação de medidas de controle da LV. 

 A identificação do vetor é um elemento chave na definição da autoctonia do caso e sugere a necessidade de monitoramento do vetor *Lu. longipalpis* com atividades de vigilância entomológica em outras localidades do município de Macapá. 
