# Supplementary material for: Primeiro registro de Lutzomyia longipalpis (Lutz & Neiva, 1912) em Macapá, Amapá
Source: Rev Saude Publica. 2024 Nov 25;58:49. [Article in Portuguese] doi: 10.11606/s1518-8787.2024058005963 (PMC11654054; doi:10.11606/s1518-8787.2024058005963)
Supplement: Translation

# Translation: The first identification of *Lutzomyia longipalpis* (Lutz & Neiva, 1912) in Macapá, Amapá

**Published:** 2024-10-21

**Authors:** Keison de Souza Cavalcante, Allan Kardec Ribeiro Galardo, José Ferreira Saraiva, Tatiane Alves Barbosa, Érika Oliveira Galeno, Marcio Claudio de Lima Nunes, Ana Paula Sales de Andrade Correa, Nayma da Silva Picanço, Fredy Galvis-Ovallos, Eunice Aparecida Bianchi Galati

**Affiliations:** I Instituto de Pesquisas Científicas e Tecnológicas do Amapá (IEPA). Laboratório de Entomologia Médica (Labenmed). Macapá, AP, Brasil; II Universidade de São Paulo. Faculdade de Saúde Pública. Programa de Pós-graduação em Saúde Pública. São Paulo, SP, Brasil; III Secretaria Municipal de Vigilância em Saúde (SMVS). Vigilância Epidemiológica/Macapá, Vigilância Ambiental, Zoonoses, Controle de Vetores, Entomologia. Macapá, AP, Brasil; IV Universidade de São Paulo. Faculdade de Saúde Pública. Departamento de Epidemiologia. São Paulo, SP, Brasil

**Keywords:** Leishmaniose Visceral, Vector, Entomological Surveillance

## Abstract

*Lutzomyia longipalpis* (Lutz & Neiva, 1912) constitutes the most epidemiologically relevant vector of visceral leishmaniasis (VL) in the New World. On October 25, 2023, the Macapá Center for Strategic Information in Health Surveillance registered a case of VL in the Km9 neighborhood, in Macapá. This study aimed to describe the Phlebotominae species in this area to assist the confirmation of the autochthony of the case. In total, 12 specimens were collected, of which five belonged to the *Lutzomyia longipalpis* species, confirming the presence of the VL agent vector and the possible autochthony of the transmission.

## INTRODUCTION

 The Phlebotominae subfamily belongs to the Psychodidae family and currently consists of about 1,060 described species worldwide ^1^ , including *Lutzomyia longipalpis* (Lutz & Neiva, 1912), the most epidemiologically relevant vector of visceral leishmaniasis (VL) agent in the New World ^2^ . It is widely present (from Mexico to Argentina), and it has been identified in all states in the Amazon region, except for the state of Amazonas ^3^ . 

 The zoonotic form of VL, caused by the protozoan *Leishmania (Leishmania) infantum chagasi* , has dogs as its main reservoir. This severe disease mainly affects children and older adults and causes high lethality in the absence of timely and adequate treatment ^4^ . 

 VL is currently spreading throughout Brazil, associated with factors such as urbanization, mobility of infected dogs, and especially vector dispersion ^4^ . Cases of VL have been identified in 25 of the 26 states of the Brazilian Federation. However, in some regions, transmission is associated with other species of Phlebotominae, which are considered secondary vectors ^4^ . The occurrence of VL has been reported in areas further east and north from the Amazon biome in Brazil, generating a debate on whether it is an enzootic disease to the region or if it has been introduced by infected migrant dogs ^5^ . 

 The municipality of Macapá was considered an area without VL transmission up to 2017, when autochthonous canine cases were reported ^6^ . Canine cases preceded human cases, depending on the presence of competent vectors in the transmission area. Despite the risk of establishing an urban cycle of transmission of this infection, few studies have been carried out on the Phlebotominae fauna in this municipality, failing to detect *Lu. longipalpis* ^6 , 7^ . 

On October 25, 2023, the Macapá Center for Strategic Information in Health Surveillance confirmed a case of VL in a 51-year-old patient who resided in the Km9 neighborhood in the aforementioned municipality. This study carried out an entomological investigation to detect Phlebotominae and assist in the confirmation of the autochthony of the case, the results of which are described below.

## METHODS

 An entomological research was conducted in the Km9 neighborhood, a peripheral area of Macapá. About 300 meters from the patient’s residence, a home shelter for about 150 dogs. The captures were carried out in the peridomicile of this property (00°03’47.82”N, 51°08’12.94”W) ( Figure ). In total, three points were selected (on both sides and at the back of the land in which the dog shelter is located) for the installation of a CDC-type light trap. The captures were carried out for three consecutive days, from November 1 to 3, 2023, totaling 108 hours of sampling. 

 The collected Phlebotominae were taken to the Medical Entomology Laboratory at the *Instituto de Pesquisas Científicas e Tecnológicas do Amapá* (IEPA – Institute of Scientific and Technological Research of Amapá) for sex screening and taxonomic identification following Galati ^3^ . All collected specimens were deposited in the IEPA zoological collection, with their respective registration numbers. 

**Figure.** Study area indicating the location of the entomological research carried out in a peripheral area of the municipality of Macapá, the Km 9 neighborhood, from November 1 to 3, 2023.

## RESULTS AND DISCUSSION

 In the three days of capture, 12 Phlebotominae individuals belonging to six species were collected in the peridomicile, namely: *Lu. longipalpis* (n = 5), *Nyssomyia antunesi* (Coutinho, 1939) (n = 3), *Micropygomyia (Sauromyia) rorotaensis* (Floch & Abonnenc, 1944) (n = 1), *Psychodopygus squamiventris maripaensis* (Floch & Abonnenc, 1946) (n = 1), *Psychodopygus ayrozai* (Barretto & Coutinho, 1940) (n = 1), and *Evandromyia (Evandromyia) brachyphalla* (Mangabeira, 1941) (n = 1) ( Table ). 

**Table.** Phlebotominae species collected in a peri-urban area in the municipality of Macapá, Km 9 neighborhood, from November 1 to 3, 2023.

**Species**	**Male**	**Female**	**Total**
*Lu. longipalpis*	1	4	5
*Ny. antunesi*	1	2	3
*Mi. (Sau.) rorotaensis*	–	1	1
*Ps. squamiventris maripaensis*	–	1	1
*Ps. ayrozai*	1	–	1
*Ev. (Eva.) brachyphalla*	–	1	1
**OVERALL TOTAL**	**3**	**9**	**12**

 Of the 12 collected specimens, five belonged to the *Lu. longipalpis* species, confirming the presence of the main vector for VL in Macapá and the local transmission of the disease. 

 The failure of previous entomological research to find *Lu. longipalpis* in the municipality ^6^^,^^7^ may be related to a recent introduction of the species in the area or to its low density, which hinders detection. However, it is worth mentioning the occurrence of *Lu. longipalpis* had been registered in Ferreira Gomes, a municipality 137 km from Macapá ^2013^ . 

 Although the occurrence of canine leishmaniasis has been reported in Macapá, the detection of a human case combined with the finding of the vector *Lu. longipalpis* in the urban area of the municipality calls for a wider awareness within health services to aid surveillance, the early diagnosis of human cases, and its timely treatment, as well as requiring measurements towards understanding the risk of infection for the human and canine population and the implementation of VL control measures. 

 Vector identification configures a key element to define case autochthony and suggests the need to monitor *Lu. longipalpis* by entomological surveillance activities in other locations in Macapá.
